# Chyluria Status Post Microwave Ablation of Renal Cell Carcinoma: A Case Report

**DOI:** 10.7759/cureus.92381

**Published:** 2025-09-15

**Authors:** Shervin Arjomand, Andrew R Sawan, James Park, Chul Chae

**Affiliations:** 1 Radiology, California University of Science and Medicine, Colton, USA; 2 Radiology, Arrowhead Regional Medical Center, Colton, USA; 3 Medical Imaging, Arrowhead Regional Medical Center, Colton, USA

**Keywords:** abdominal radiology, chyluria, fat-fluid level, radiofrequency ablation (rfa), renal cell carcinoma (rcc)

## Abstract

Microwave ablation is a minimally invasive alternative to nephrectomy for select patients with renal cell carcinoma (RCC), particularly those with solitary kidneys or significant comorbidities. We present a case of a 55-year-old male with stage 3B chronic kidney disease and biopsy-confirmed clear cell RCC who underwent successful microwave ablation. Post-procedure imaging demonstrated a stable lesion size without recurrence. However, follow-up revealed persistent chyluria, likely as a rare and delayed consequence of ablation. This report discusses the pathophysiology, diagnostic considerations, and clinical implications of chyluria in the post-ablation setting.

## Introduction

Chyluria is defined as the leakage of lymphatic fluid into the urinary tract, resulting in the passage of milky white urine. It is most common in tropical regions of Southeast Asia, where lymphatic filariasis remains endemic. WHO data from 2023 estimate that 40 million people are infected with lymphatic filariasis, with chyluria occurring in approximately 2% of those affected [1,2]. In this setting, the parasite infiltrates the lymphatic system surrounding the bladder, causing obstruction and subsequent leakage [3]. In contrast, chyluria is rare in Western countries and is typically associated with iatrogenic causes. Reports describe its occurrence after partial nephrectomy, with one series noting 4 cases among 125 patients, and additional case reports documenting similar presentations [4-6]. Thermal ablation procedures have also been implicated, with one retrospective study showing chyluria in 41% of patients following radiofrequency ablation, in addition to case reports of post-ablation chyluria [7,8]. To date, only a single case report has described chyluria following microwave ablation, underscoring the rarity of the present case [9]. The underlying mechanism is believed to be the formation of a lymphatic-urinary fistula secondary to surgical or ablative disruption. Imaging plays a critical role in diagnosis, with CT demonstrating a fat-fluid level in the bladder that is highly suggestive of chyluria [8]. Laboratory confirmation relies on detecting chylomicrons and triglycerides in urine [10]. This case highlights chyluria as a rare but important long-term sequela of renal tumor ablation, emphasizing the need for awareness among clinicians managing patients after thermal interventions.

## Case presentation

**Figure 1:**
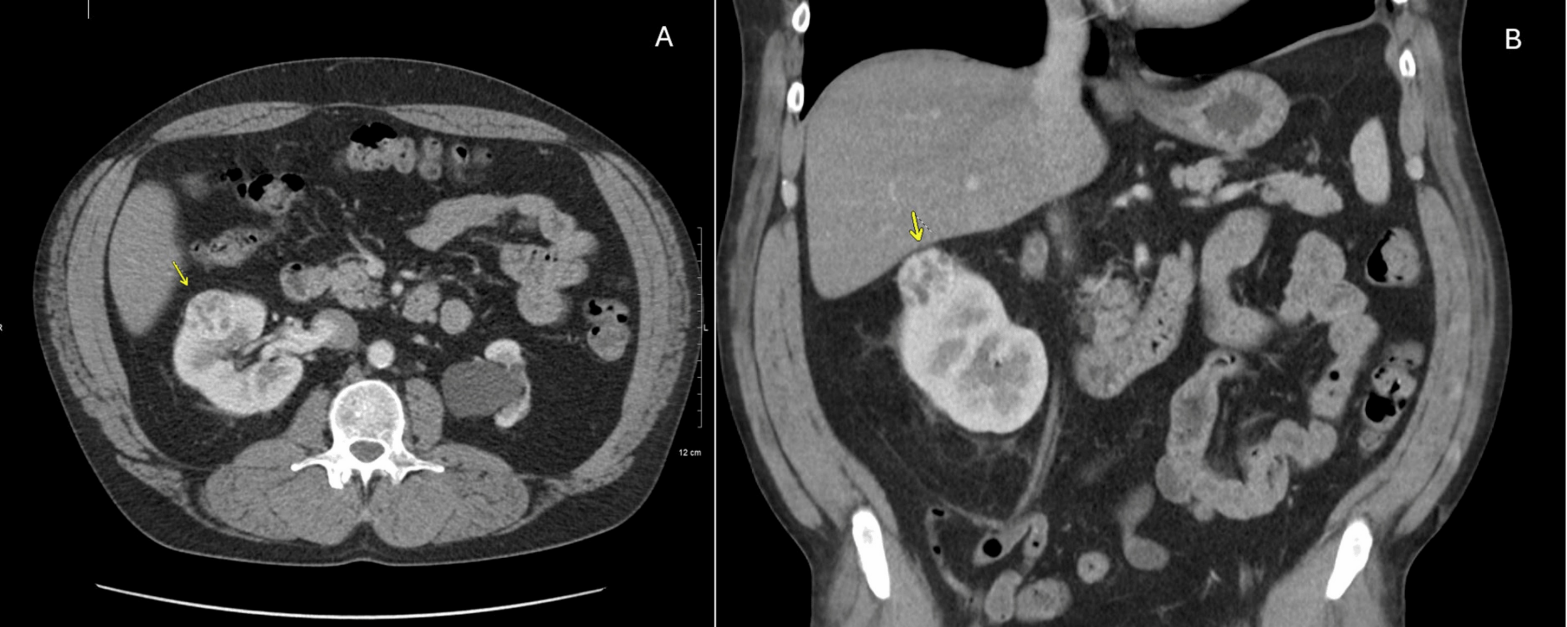
Axial, CT A/P w/ IV contrast (image A) and Coronal, CT A/P w/ IV contrast (image B) A 3.4 cm cortical mass is shown on axial (image A) and coronal (image B) views, with yellow arrows pointing to the mass. A/P: abdomen/pelvis

A 55-year-old male with a past medical history of stage 3b kidney disease with no significant symptoms was referred to nephrology to manage the patient’s chronic kidney disease. Initial workup included a renal ultrasound, revealing an incidental mass found on US, leading to a follow-up CT that revealed a 3.4 cm cortical mass in the right kidney (Figure 1).

**Figure 2:**
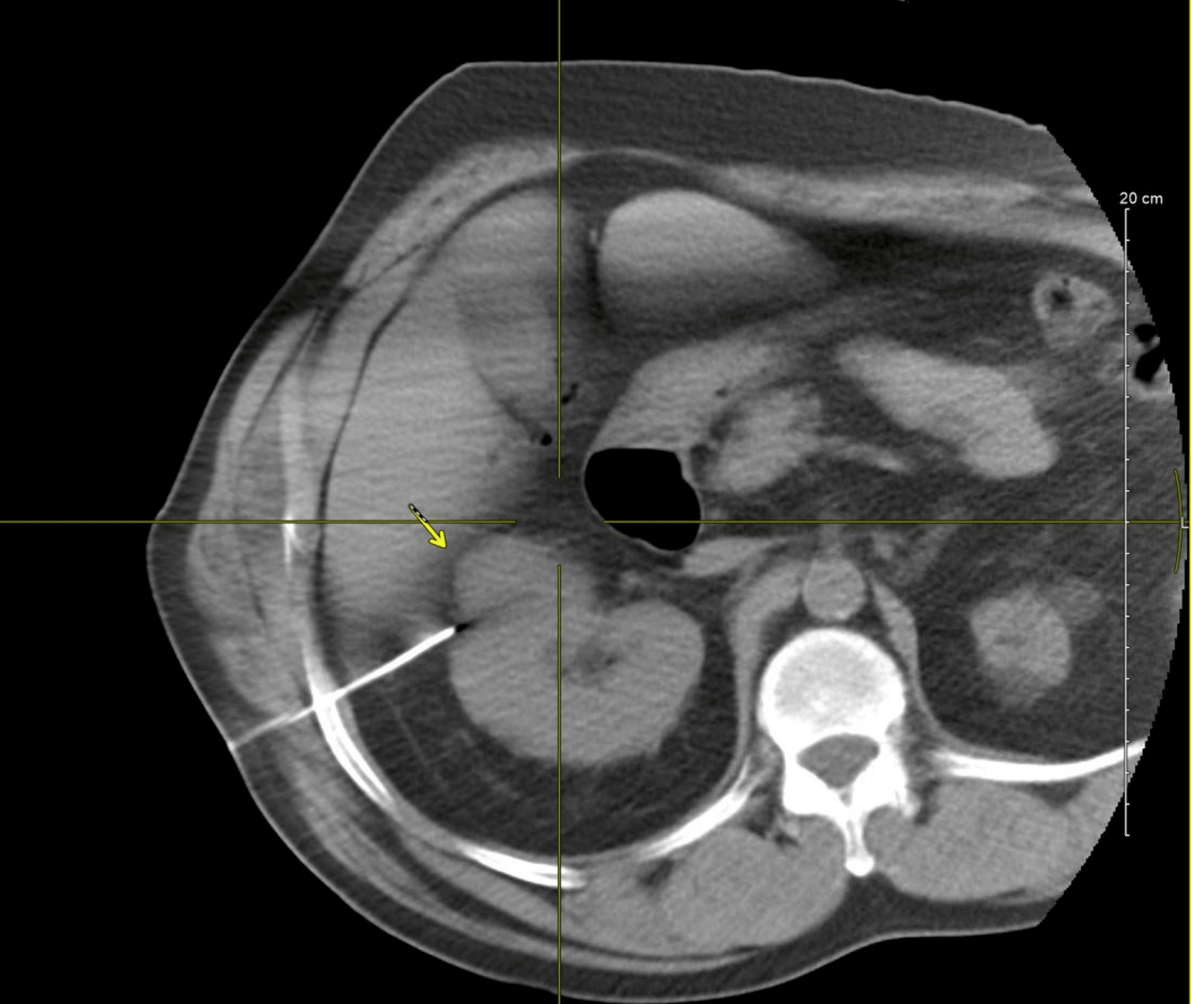
Axial, IR-guided thermal ablation Using ultrasound guidance, a 16-gauge guiding needle was advanced to 3 cm from the margin of the renal mass, and microwave ablation was performed at 60W for 15 minutes with no immediate hemorrhage or other complications. Extensive hydrodissection was done prior to ablation, performed using the guiding needle to separate the colon from the kidney, while approximately 150 cc of 1% contrast in the 5W was injected to separate the colon from the kidney. IR: interventional radiology

Given comorbidities and tumor characteristics, the patient underwent percutaneous microwave ablation, consistent with the American Urological Association and American Society of Clinical Oncology guidelines, which support thermal ablation as an alternative to partial nephrectomy for small renal masses in select patients [11-13]. Using ultrasound guidance, a 16-gauge guiding needle was advanced to the posterior margin of the mass, with needle location confirmed by CT. Biopsy was performed initially using a coaxial 18-gauge needle, with a single specimen obtained. Extensive hydrodissection was then performed using the guiding needle to separate the colon from the kidney, with approximately 150 cc of 1% contrast in the 5W was injected to separate the colon from the kidney. A 14-gauge 20 cm AMICA probe was then advanced into the renal mass using CT guidance, with an additional hydrodissection with 200 cc of diluted contrast used. After hydrodissection, the needle, at least 3 cm from the margin of the renal mass, was used to perform microwave ablation at 60W for 15 minutes with no immediate hemorrhage or other complications (Figure 2). Histopathology confirmed clear cell RCC, International Society of Urological Pathology (ISUP) Grade 1.

**Figure 3:**
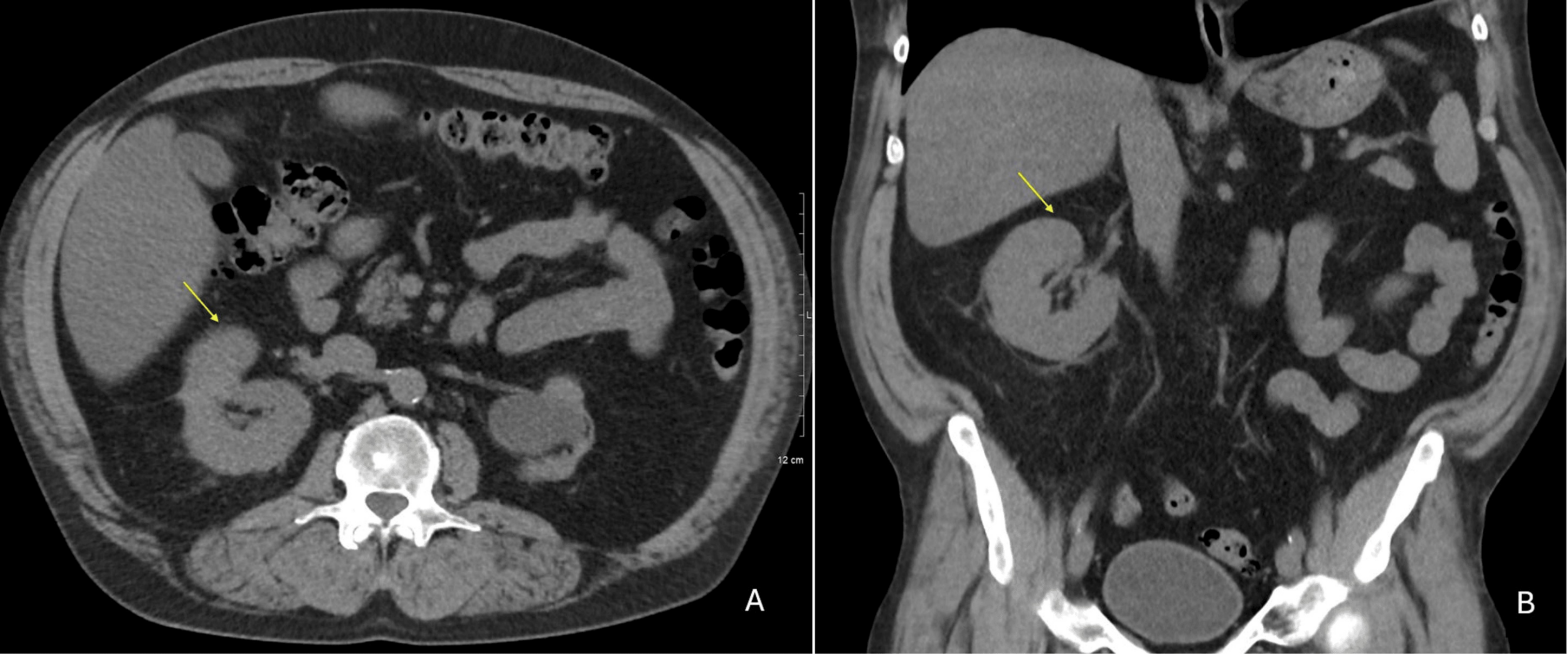
Noncontrast CT A/P S/P ablation of right kidney, axial (image A) and noncontrast CT A/P S/P ablation of right kidney, coronal Noncontrast CT abdomen/pelvis (A/P) status post (s/p) ablation of the right kidney around five years after microwave ablation. The mass is difficult to visualize on coronal (image B, yellow arrows) but can be seen on axial (image A, yellow arrows). Mass size decreased from 3.4 cm to 2.5 cm and has been stable in size.

**Figure 4:**
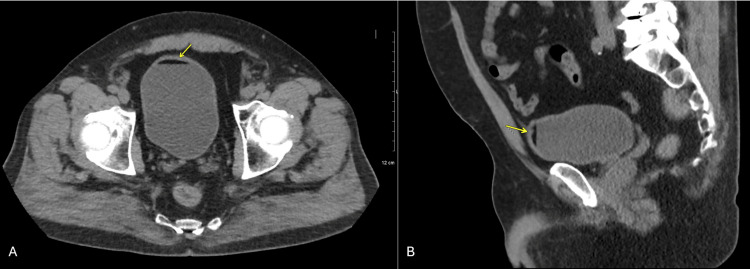
Follow-up CT with chyluria, axial (image A) and sagittal (image B) Fat-fluid level highlighted with yellow arrows on both axial (image A) and sagittal (image B), pathognomic for chyluria

**Figure 5:**
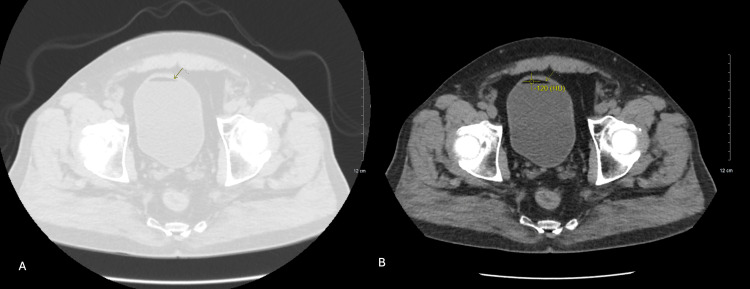
Follow-up CT, axial view, in the lung window (A) and with HFU measurement (B) The lung window (image A) authenticates that the radiolucency in the bladder is not air, and the Hounsfield unit (HFU) measurement of -120 (image B) verifies the lipid-like fluid, confirming chyluria.

Post-op recovery was uneventful, with immediate post-op pain managed with one low-dose IV Dilaudid and 500 mg Tylenol. The patient endorsed mild right flank pain, non-radiating and stable in character, which did not impact function, but no episodes of urinary tract infection or hematuria post-operation were noted, and no gross hematuria, dysuria, fevers, or weight loss were reported during long-term follow-up. Routine imaging of the mass has been done biannually to track the size of the mass, which decreased from 3.4 cm to 2.9 cm immediately post-ablation, then down to 2.5 cm on further follow-up imaging (Figure 3). Additionally, follow-up CT imaging five years after the ablation reveals a fat-fluid sign, indicating chyluria (Figure 4). A lung window (Figure 5, image A) authenticates that the radiolucency in the bladder is not air, and the Hounsfield unit (HFU) measurement of -120 (Figure 5, image B) verifies lipid-like fluid, confirming chyluria [14]. However, there is no biochemical evidence, as during follow-up for five years, the patient remained asymptomatic without milky urine. Urinalysis showed no chylomicrons or triglycerides.

## Discussion

Microwave ablation of a renal mass can cause chyluria through the formation of a lymphatic-urinary tract fistula, most likely due to thermal injury to perirenal lymphatic vessels. The high temperatures generated during ablation can disrupt the integrity of these lymphatics, which are anatomically adjacent to the renal parenchyma and collecting system, allowing chyle to leak into the urinary tract and present as milky urine. Furthermore, the subsequent inflammatory response and tissue necrosis may promote local tissue remodeling and persistent fistulous communication between the lymphatic and urinary systems, perpetuating chyluria. While this complication is rare, similar mechanisms have been described following other renal interventions such as partial nephrectomy and radiofrequency ablation, supporting the biological plausibility of this process after microwave ablation as well [4-7]. Notably, the presence of fat-fluid levels within the bladder on CT imaging supports the diagnosis [8]. Extensive evaluation revealed no evidence of parasitic infection, trauma, or recurrent malignancy as an alternative etiology. The patient’s clinical course has remained benign, and chyluria has not required intervention. This case illustrates a rare but benign long-term complication of renal microwave ablation. Supporting this finding is that, to our knowledge, there has only been one case study published documenting chyluria after microwave ablation [9]. Our patient did not have chyluria identified on imaging until five years after ablation, which is longer than the two years in the previous case study before identification [9]. Interestingly, both our patient and the one in the other study were completely asymptomatic, with no lipids found in urinalysis [9]. 

This unique pathology has been documented more frequently for other renal interventions such as radiofrequency ablation and partial nephrectomy. A retrospective study discussed above demonstrates a chyluria incidence rate of 41% in a small population (17/41) of people who underwent RF ablation [4]. While this article includes a small sample size to draw any true incidence from, it does suggest that chyluria occurs more often after RF ablation versus cryoablation and microwave ablation. Furthermore, chyluria after partial nephrectomy is described in case studies and one study that found 4 cases of chyluria in 125 patients who had undergone partial nephrectomy, making chyluria after partial nephrectomy a rare but recognized complication [6-8]. For cryoablation of renal masses, there are no published reports that recognize a correlation with chyluria despite large systematic reviews of adverse events associated with renal cryoablation, suggesting the incidence is extremely low or negligible [15].

Chyluria may go unrecognized if not radiographically identified or if urine is not grossly milky, as in this case. While typically asymptomatic, chronic chyluria can rarely lead to protein loss, hypolipidemia, or urinary obstruction, none of which occurred in this patient. Chyluria after renal ablation may resolve spontaneously or with conservative management such as dietary modification to reduce long-chain triglyceride intake [16]. In most cases, intervention is not required unless symptoms are severe or persistent, in which case sclerotherapy or surgical lymphatic disconnection may be considered [6,17-19]. In this patient, chyluria was self-limited and did not require invasive intervention.

## Conclusions

Microwave ablation is an effective treatment for small renal masses in patients with limited renal reserve, but clinicians should be aware of rare delayed chyluria detectable on imaging, even in the absence of procedural complications. Should a patient begin to experience symptoms such as milky white urine, monitoring patients' labs to look for protein loss, hypolipidemia, and urinary tract obstruction are important. Key findings on post-ablation imaging showing fat-fluid levels within the bladder.
